# The opportunities and challenges of an Ebola modeling research coordination group

**DOI:** 10.1371/journal.pntd.0008158

**Published:** 2020-07-16

**Authors:** Caitlin Rivers, Simon Pollett, Cecile Viboud

**Affiliations:** 1 Johns Hopkins Center for Health Security, Maryland, United States of America; 2 Viral Diseases Branch, Walter Reed Army Institute of Research, Marlyand, United States of America; 3 Uniformed Services University of the Health Sciences, Maryland, United States of America; 4 Marie Bashir Institute, University of Sydney, New South Wales, Australia; 5 National Institutes of Health, Maryland, United States of America; Institute for Disease Modeling, UNITED STATES

In response to the protracted Ebola virus outbreak in the Democratic Republic of Congo, the international public health community called for increased attention, coordination, and resources to support the response. The use of real-time modeling and analytics to support public health decision-making (also known as “outbreak science”) has been an important capability that has grown during previous outbreaks [1–3]. Despite the informative role that infectious disease models played in the recent DRC outbreak [4–7], cross-talk within the infectious disease modeling community and between infectious disease modelers and model stakeholders, such as health agencies, may be limited. Lack of communication can reduce the potential use of modeling capability to inform outbreak prevention and mitigation strategies. For example, mathematical modelers may not be aware of questions that would be particularly useful for guiding the response, such as the location and staffing of Ebola treatment units. On the other end, public health teams may not be aware of outbreak features that may signal improvement or worsening of incidence or increased potential for spatial spread.

**Fig 1:**
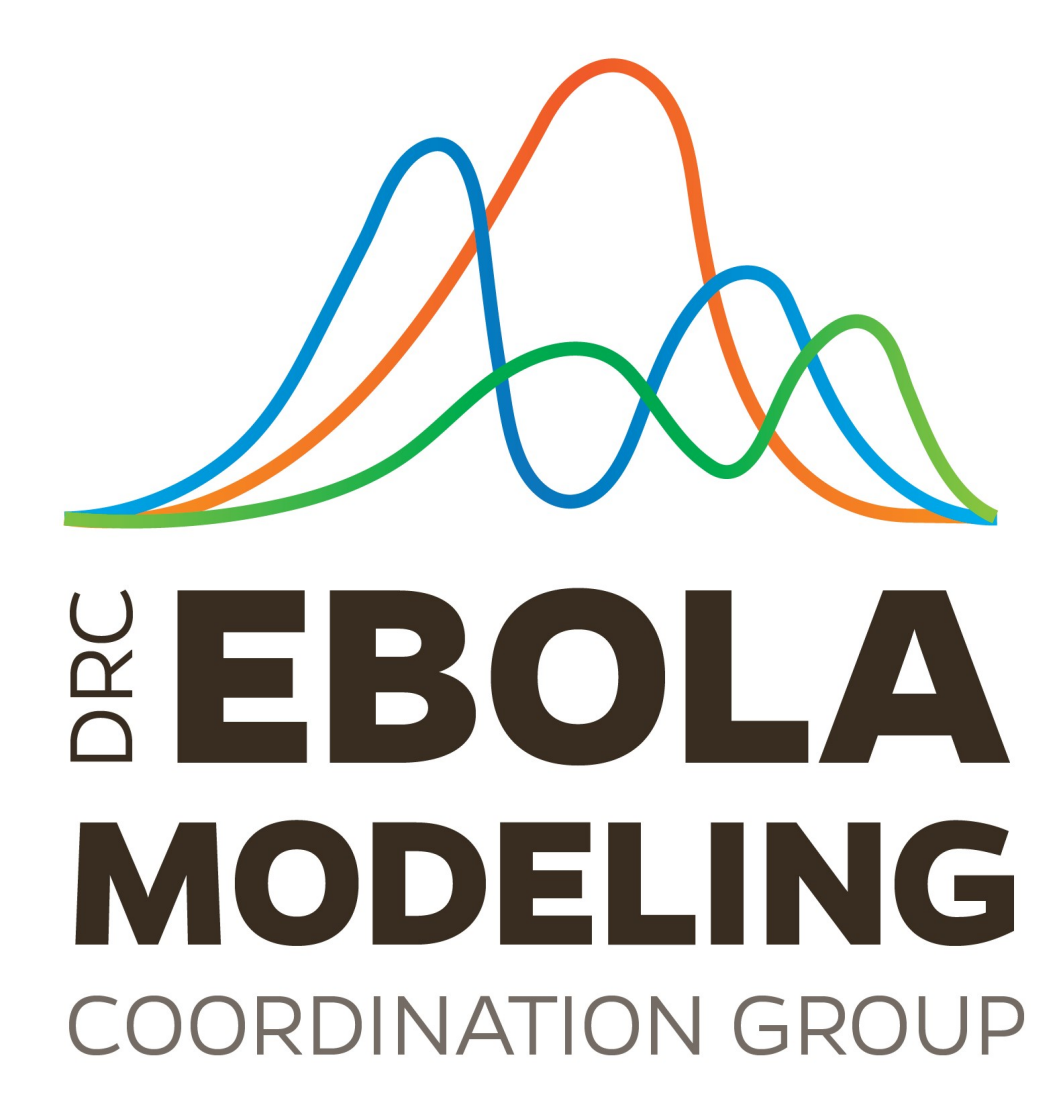
The logo of our informal Ebola modeling coordination group. DRC, Democratic Republic of Congo.

To improve communication and create awareness of the efforts of modeling groups already studying the DRC Ebola outbreak, we—an existing outbreak science working group—convened an informal modeling research coordination group to align efforts around infectious disease modeling of the outbreak (Fig 1). Each month, two modeling teams were invited to present their preliminary research to the group via video conferencing. Participants were invited to ask questions, make suggestions and critiques, and share ideas. Broader discussion of current challenges and open questions in directing the response were encouraged. Over 170 participants from governments, academia, and nongovernmental organizations from around the world signed up to receive invitations to the meeting. Approximately 40 stakeholders were on any given call, including those with Ebola response activities. Teleconference calls were supplemented by communication through a Slack-enabled virtual workspace, public and private GitHub repositories, and a mailing list. The emphasis on this modeling coordination framework was to facilitate scientific research exchange.

The presentations throughout the seven months of this modeling coordination group meetings highlighted some of the ways this DRC outbreak differed to the prior 2014 West African outbreak. The impact of violence itself on epidemic growth and effective response was also a major focus of modeling efforts [8]. Shifts and development of data sources since 2014 West African modeling efforts were also conspicuous, particularly in the domain of social sciences. For example, social media mining was used to measure community sentiment and estimate how this may impact Ebola response and countermeasure implementation [9]. Socio-economic, demographic, and environmental data were used in ecological models which predicted the location and risk of future Ebola epidemics in Africa [10]. The group heard perspectives of an anthropologist who provided lessons learned on the social context of Uganda Ebola outbreak responses, of major relevance to DRC Ebola models. While genomic modeling played a key role in the 2014 West African epidemic, presentations at this modeling group indicated how these tools had matured with inclusions of interpretative narratives accompanying real-time phylogenies to improve their interpretation and use by field epidemiologists [11]. Unlike the prior West African epidemic, licensed Ebola vaccines were available and were implemented as a licensed product countermeasure, and models have played a key role in guiding vaccination strategies and vaccine demand [12].

This working group was not formally endorsed by any agency or formally tasked with supporting any specific public health activity in the DRC. It was not intended to replace formal modeling groups who had already been tasked to support the Ebola response and is distinctly different from epidemic surveillance sharing platforms such as the Global Public Health Intelligence Network or the Epidemic Intelligence Information System [13,14]. However, we propose that this community forum was complementary to those efforts and served several valuable purposes. It allowed early modeling results to be shared with other modelers and model stakeholders, with the goal of enabling more efficient and effective infectious disease modeling relevant to this particular outbreak. Although preprint servers and other rapid publication venues are useful for sharing relatively early modeling results, there are still consequential delays between the generation and reporting of results, making them of limited use in the field [15,16]. The DRC modeling coordination group aimed to minimize this gap by providing a unique opportunity for discussion of prepublication results and provides a platform for data sharing where feasible. It also facilitated awareness about the most important and timely public health questions that modelers and other researchers could help to address to reduce duplication and ensure that modelers were answering the right questions at the right time. Finally, it allowed resources such as open access data and parameter estimates to be shared, thereby improving model quality, comparability, and efficiency of the modeling process.

The Ebola modeling coordination group also highlighted the pervasive complexities of conducting research during epidemics. For example, there is a need to balance broad participation of scientists to study and help resolve the crisis while also deferring to the more urgent priorities of health authorities who are engaged in the critical public health response as well as respecting existing local and international research efforts already formally tasked with supporting the outbreak. It is also a priority to conduct equitable research by collaborating with locally-based scientists so that they are properly involved in and credited with the international scientific response, including data collection efforts. We propose that coordination groups, like the one described here, could help with that. Moreover, we have demonstrated how this model coordination framework could readily be adopted by other groups for future epidemics and indeed has been so in the current COVID-19 outbreak [17].
